# Surface Stoichiometry and Roughness of a Degraded A380 Alloy after Casting, Technical Cleaning and Packaging

**DOI:** 10.3390/ma14216458

**Published:** 2021-10-28

**Authors:** Darja Steiner Petrovič, Djordje Mandrino

**Affiliations:** IMT, Institute of Metals and Technology, Lepi pot 11, 1000 Ljubljana, Slovenia; darja.steiner.petrovic@imt.si

**Keywords:** Al–Si, die casting, technical cleaning, detergent, humidity, corrosion, oxide, hydroxide, surface roughness, X-ray photoelectron spectroscopy

## Abstract

The surface stoichiometry of the degraded surface of a commercial Al–Si casting was investigated. The die-cast component was previously stored in a sealed plastic envelope. After that, surface stains in the form of white layers were observed. X-ray photoelectron spectroscopy (XPS) was used to study these layers. For comparison, a seemingly unaffected area as well as a freshly cut surface of the casting were also analysed. In order to additionally assess the surface condition, surface roughnesses were measured. Based on the binding energies (BEs) of the Al and O in the XPS spectra, and the stoichiometric results, it was concluded that the surface layers of the degraded and undegraded samples consist mostly of aluminium oxide and aluminium hydroxide. Furthermore, sodium phosphate from the leftover detergent and silicon oxide were detected in both analysed areas. Analyses of the Al KLL Auger transition were used to corroborate this. The relative shares of Al oxide vs. hydroxide based on the elemental concentrations were determined. The chemical compositions and chemical states of the elements in the top layers were thus obtained. The combination of surface-sensitive analytical techniques was found to be a suitable tool for the ex-post identification of the source of defects.

## 1. Introduction

When producing suitable and reliable Al castings, the formation of oxide films during the metal’s melting, delivery, transfer, and casting must be avoided. In addition, achieving a flawless surface treatment of the castings remains a great challenge for the metal foundry industry [1]. Dedicated studies aiming to improve the castability of Al–Si alloys [2,3], their mechanical properties [4], the properties of wear and friction [4,5,6,7,8], aging and fatigue behaviour [9,10,11], fouling and corrosion resistance [12,13], etc., are extensive and constantly ongoing.

It is well-known that the lifespan of castings decreases due to sticky sand and surface roughness [14]. It is necessary, therefore, to clean the castings to a certain cleaning value, with respect to the lead time [14]. The technical cleaning of Al alloys is one of the major problems in manufacturing because of the difficulty in preventing subsequent corrosion [15,16]. Cast aluminium components are usually cleaned in a prescribed sequence of operations to achieve the required standard of technical cleanliness. Therefore, reporting good practices [14,17] is essential for the foundry community to ensure the exchange of information on optimal, stain-free, and compliant cleaning procedures, in accordance with the highest environmental standards. Tiringer et al. systematically studied the influence of mechanical (grinding and polishing) and chemical (cleaning) treatments on the morphology and surface composition of selected Al-Zn-Mg and Al-Cu-Mg alloys [18]. It was shown that both treatments have strong effects on the chemical composition, morphology, and wettability of the surfaces. Consequently, the corrosion properties of these alloys are altered. Undoubtedly, the processes occurring during chemical cleaning are complex and specific for each Al alloy [18].

There is a naturally formed oxide layer with a thickness of about several nanometres on the surface of an Al alloy [19,20,21]. This native oxide layer is highly defective and cannot provide effective protection from corrosion, especially when different types of heterogeneities are incorporated within it [16,19,20,21,22]. Therefore, the corrosion resistance of cast Al–Si alloys strongly depends on the surface condition, predominantly on the silicon content and the surface roughness [23,24]. The surface roughness has a significant effect on reducing the corrosion resistance, as does a change in the surface chemistry. Here, the constant trade-off between these two influential surface parameters must be considered [13,23,24].

On the other hand, further corrosion studies are needed to confirm whether the contact angle is a significant factor in the variation of corrosion resistance [23]. A recent study demonstrates that both the morphological characteristics and the chemical composition of the surface mutually contribute to the hydrophobicity of Al alloys, which must be taken into account when anti-friction, anti-wear, and corrosion-resistant Al alloys are developed [25].

The surface topographies of many kinds of industrial products are usually created in the final stages of production. Surface roughness measurements are of great importance in automotive and other metal-work industries [26,27].

Different techniques of surface roughness measurements and various instruments are available. In general, the measurement techniques can be divided into two broad categories, the contact type and the non-contact type, while measuring methods may be divided into six categories: mechanical stylus method, optical methods, scanning-probe microscopy methods, fluid methods, electrical methods, and electron microscopy methods [26,27,28,29].

In the field of cast Al–Si alloys, wear behaviour is of special importance, since the material is one of the most important engineering alloys [4,5]. For cast piston Al–Si alloys, depending upon the lubrication conditions, wear types can be adhesion, abrasion, and cavitation. After a long sliding time, factors such as the average coefficient of friction, surface roughness, depth of the deformed layer, and composition and microstructure of near-surface material tend to control the wear [5]. A eutectic Al–Si alloy exhibits a larger running time to steady-state friction conditions with an increase in the surface roughness parameters under both dry and oil-lubricated sliding conditions [6]. In hypereutectic Al–Si alloys, the improvement in the wear resistance is mainly attributed to the decreasing size of the primary Si phase and the corresponding surface roughness [7]. It has been claimed, that, in general, hypoeutectic Al–Si alloys have a higher wear resistance than hypereutectic alloys [4].

Due to the increased need for applications that require high strength and wear resistance, additions of various alloying elements, e.g., Mg, Cu, and Zn on the microstructure, and mechanical and tribological properties are investigated [4,30], and parallel to this, additions of various particles in the case of Al-based, metal-matrix composites (MMCs) [13,24]. For an assessment of the wear behaviour, machine-learning models currently available are a very supportive tool in predicting the friction and wear of Al alloys from material and tribological test data [7,8,13,24]. A proper topography characterization and further calculations of the representative surface parameters are essential to correlate manufacturing process parameters and key quality factors. Consequently, the functional properties of finished elements can be predicted, and the design of better-optimised topographies is enabled [2,10,11,31,32,33,34].

For an improvement in the tribological behaviour of contact surfaces, the easiest way without changing the geometry is by changing the surface roughness [34]. Surface roughness parameters, i.e., skewness (Ssk) and kurtosis (Sku), show a good correlation with the tribological performance of contact surfaces. Surfaces with higher Sku and negative Ssk values tend to reduce friction [35].

The present study addresses the undesirable surface degradation of a commercial casting made of the widely used aluminium die-casting alloy A380. The die-cast component was finished according to the prescribed technological route, packaged, and shipped from the producer to a customer.

The study elucidates the surface stoichiometry of the degraded surface of a finalized Al–Si casting. The surface-sensitive analytical technique x-ray photoelectron spectroscopy (XPS) was used to analyse the degraded and non-degraded parts of the surface. These were compared to measurements performed on a pristine, freshly cut area. In order to assess the surface condition, the surface roughness was measured by high-resolution 3D optical microscopy. The topography was evaluated using various statistical parameters of the surface roughness. In addition, calculated relations between the stoichiometry and the relative shares of Al oxide vs. Al hydroxide were used to determine the share of Al_2_O_3_ present on various surfaces of the casting.

The present study refers to the use of an unconventional combination of complementary, surface-sensitive techniques, i.e., XPS and a high-resolution 3D optical microscopy, to identify the source of the defects on the cast components.

## 2. Materials and Methods

### 2.1. Visual Examination and Optical Imaging

Visual inspection and optical imaging of the surface were performed using an Olympus stereo microscope (Olympus, Hamburg, Germany). A geometrically similar sample was also taken from a freshly cut area of the material for a comparison with the degraded surface.

### 2.2. Material

The casting under investigation was manufactured by industry from an A380 aluminium alloy with the chemical composition specified in Table 1. This aluminium–silicon alloy is generally used for automobile engine parts, especially cylinder heads [3,4,11,36]. The chemical composition was determined by inductively coupled plasma optical emission spectroscopy (ICP-OES) using an Agilent 720 instrument (Agilent Technologies, Inc., Santa Clara, CA, USA).

### 2.3. Sampling

#### 2.3.1. XPS Analysis

**Figure 1:**
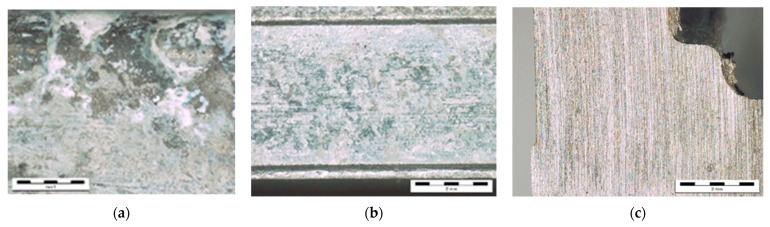
Stereo-microscope images of the degraded surface of the Al–Si alloy (**a**), undegraded area (**b**), and cross-section of the freshly cut casting. (**c**) Magnification: 20×.

Representative samples for the XPS analyses were taken from the (i) degraded and (ii) non-degraded areas according to the criterion of the most (least) deteriorated surface. Area for the freshly cut cross-section of the Al–Si casting was chosen randomly. Samples were named according to the sampling sites. The analysed areas were approximately 3 × 3 mm^2^ in size (Figure 1).

#### 2.3.2. Surface Roughness Measurements

Surface roughness measurements were performed on the same three samples already selected for XPS analyses. For clarity, approximately twice the area of the degraded sample shown in Figure 1a was analysed.

### 2.4. XPS Analysis

Surface analyses were performed using a Microlab 310F X-ray photoelectron spectroscopy (XPS) instrument (VG Scientific Ltd., East Grinsted, UK). The Cu–K_α_ line at 1253.6 eV and the Al–K_α_ line at 1486.7 eV were used for the XPS. An anode voltage and emission current of 12.5 keV × 16 mA = 200 W were used. This is within the power range usual for this type of measurement (100–400 W). After a first survey spectrum, several minutes of argon-ion (Ar^+^) etching were applied with an ion-beam energy of 3 keV and an ion current of 0.8 µA. This etched away the topmost several nanometres of carbon contamination. Avantage 3.41v software, provided by the instrument manufacturer, was used for the data acquisition and processing of the surface-spectroscopy results. In addition, commercially available Casa XPS software (Version 2.3.15, Casa Software Ltd., Teignmouth, UK) [37] was used for the data processing.

### 2.5. Surface Roughness Measurements

The surface roughness of the samples was measured using a high-resolution 3D optical microscope Alicona InfiniteFocus G4 3D (Alicona Imaging GmbH, Raaba bei Graz, Austria) based on a variation of the focus, intended for topography and form measurements. For the cast surfaces, 3D surface parameters give a more accurate reflection of the surface and are more precise than their 2D counterparts [29].

Measurements were performed at a magnification of 50×. Due to the very high contrast between the surface constituents, a 15-nm-thick carbon overlay was sputtered on top of Sample 1 using a precision etching coating system (Gatan 682, PECS instrument, GATAN/AMETEC GmbH, Unterschleissheim, Germany) to achieve an acceptable contrast surface for the measurements.

## 3. Results

### 3.1. Imaging of the Surfaces

Before the surface analysis, a visual inspection of the surface was performed using stereomicroscopy. A representative area of the degraded surface is shown in Figure 1a. For comparison, samples for the XPS analyses were also taken from the undegraded area of the casting (Figure 1b) and from the freshly cut bulk material (Figure 1c).

### 3.2. XPS Surface Analysis

#### 3.2.1. Surface Chemistry of the Degraded Areas

**Figure 2:**
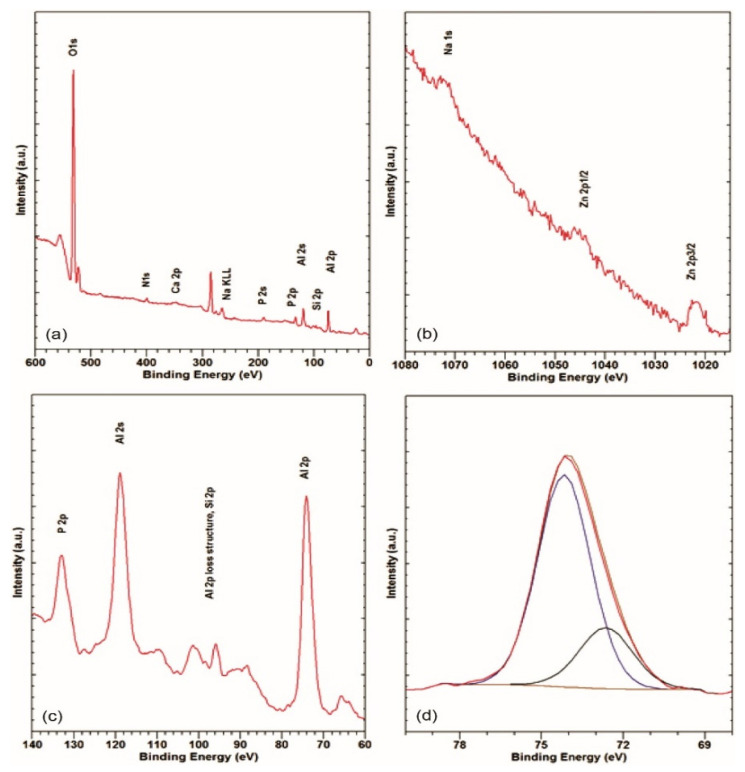
XPS survey (**a**), region around Na 1s, Zn 2p (**b**), 60–140 eV region (**c**), and narrow-range Al 2p (**d**) spectra in the degraded area.

XPS analyses were carried out on the degraded areas presented in Figure 1a. The XPS spectra are shown in Figure 2a–d.

The survey spectrum from the degraded area is shown in Figure 2a. It shows strong Al 2p, Si 2p, Al 2s, C 1s, P 2p, and O 1s peaks. The low-intensity Ca 2p at a binding energy (BE) of approximately 347 eV and the Ar 2p at approximately 242 eV can be seen. The N 1s at approximately 400 eV, corresponding to nitrogen in an organic matrix, can be observed [38,39]. There is also a small peak at around 465 eV due to the K_α_-induced O 1s transition [38,39]. Figure 2b shows the *BE* region of 1015–1080 eV, where the highest-intensity Na and Zn peaks, i.e., Na 1s, Zn 2p_3/2_, and Zn 2p_1/2_, that are barely visible in the survey spectrum can be observed. The Zn 2p_3/2_ at an approximate *BE* of 1022 eV suggests oxidized zinc. The Na 1s at approximately 1072.0 eV is compatible with Na in the phosphates used in detergents. In Figure 2c, the *BE* region between 60 and 140 eV measured at a higher resolution is shown. The Al 2p and Al 2s peaks at approximately 73.5 and 119.0 eV suggest an Al oxide surface. A low-intensity Al 2p loss structure appears between 80 and 105 eV [39,40]. This is probably due to the obvious inhomogeneity of the surface (Figure 1a), where small, undegraded areas of the Al–Si alloy, or more probably, such areas under a very thin oxide layer, might remain.

The loss structure was fitted to obtain an estimate of the Si 2p intensity, since they overlap. In Figure 2d, a narrow range of the Al 2p XPS spectrum is shown, where the asymmetry of the peak suggests non-negligible, lower-*BE* components.

Fitting yields Al 2p components at 72.7 eV, corresponding to elementary aluminium, and 74.2 eV, corresponding to Al oxide or hydroxide [38,39]. Thus, on the degraded area of the sample, around 25 at% of unoxidized Al can still be found. The P 2p at approximately 133 eV (Figure 2a,c) corresponding to phosphate is also observed, which is consistent with the leftover detergent, as already suggested by the Na 1s. The BE values of the peaks and atomic concentrations of the elements, derived from the survey and the narrow range spectra, are summarized in Table 2.

#### 3.2.2. Surface Chemistry of an Undegraded Surface

**Figure 3:**
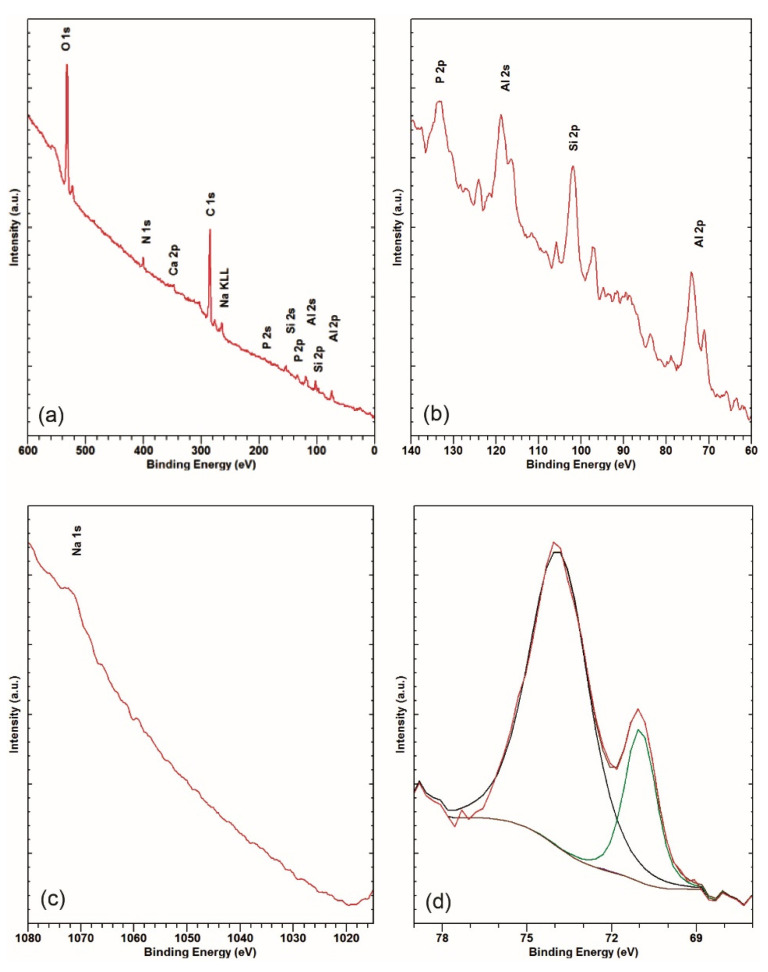
XPS survey (**a**), 60–140 eV region (**b**), region around Na 1s, Zn 2p (**c**), and narrow-range Al 2p (**d**) spectra in the undegraded area.

The survey spectrum measured on the undegraded area in Figure 3a shows strong Al 2p, Si 2p, Al 2s, C 1s, P 2p, and O 1s peaks.

On closer examination, the Ca 2p at approximately 347 eV and the Ar 2p at approximately 242 eV can be seen. The N 1s at approximately 400 eV corresponding to nitrogen in an organic matrix can be observed [38,39]. There is also a small peak at around 465 eV due to the K_α_-induced O 1s transition [38,39]. Figure 3b shows the BE region between 60 and 140 eV measured at higher resolution. It is clear that the Al 2p and Al 2s peaks consist of two components, suggesting a metallic and an oxide/hydroxide phase. The Si 2p peak at approximately 101.0 eV, suggesting sub-stoichiometric Si oxide, can be observed. The P 2p at approximately 133 eV (Figure 3a,b) corresponding to phosphate is also observed. This is consistent with the leftover detergent, also suggested by the Na 1s, clearly observed in the *BE* region of 1015–1080 eV (Figure 3c). Zn 2p_3/2_ and Zn 2p_1/2_ at approximately 1022 eV and Zn 2p_3/2_ at approximately 1045 eV are, however, not observed. In Figure 3d, a narrow-range Al 2p XPS spectrum is shown. Fitting it with metallic and oxide/hydroxide components is an obvious choice, i.e., Al 2p components at 72.3 eV, corresponding to elementary aluminium, and 73.9 eV, corresponding to Al oxide or hydroxide [12,13,14], are thus obtained.

#### 3.2.3. Surface Chemistry of the Freshly Cut Material

**Figure 4:**
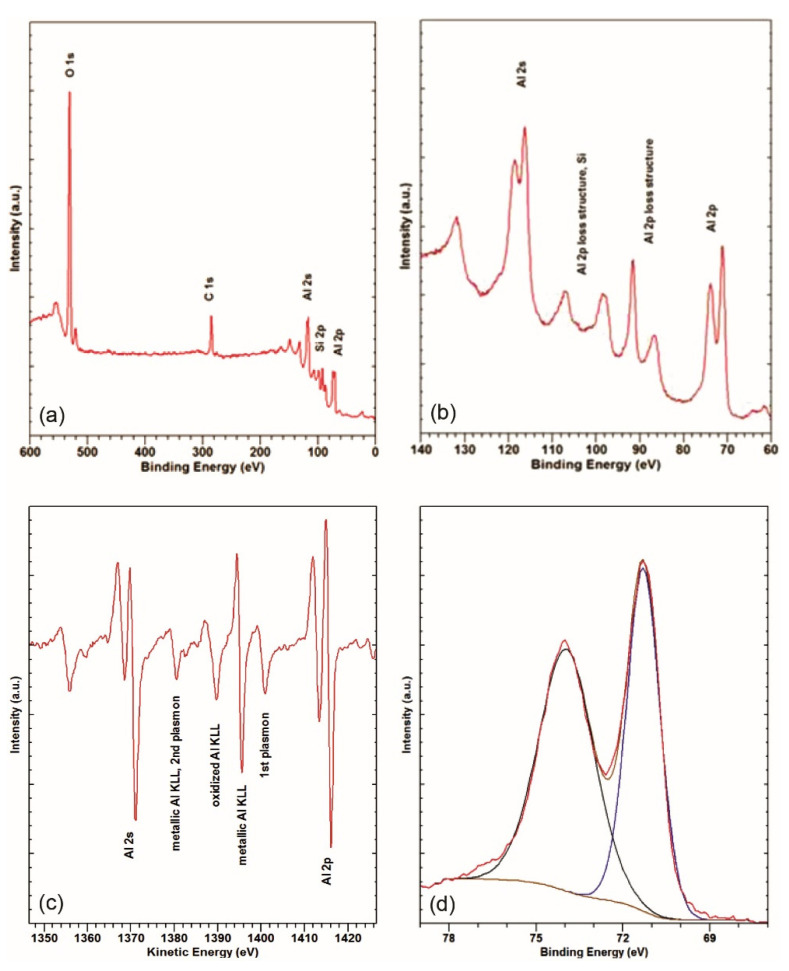
XPS survey spectrum (**a**), narrow-range 60–140 eV region (**b**), differentiated XPS spectrum from the 60–140 eV region (**c**), and narrow-range Al 2p (**d**) measured on the freshly cut surface.

For comparison, an XPS analysis of the freshly cut surface represented in Figure 1c was performed (Figure 4).

The survey spectrum of the freshly cut surface in Figure 4a shows strong Al 2p, Al 2s, C 1s, and O 1s peaks. Ca 2p and Ar 2p do not appear, since this surface was exposed to the atmosphere for a short time (minutes) and was not ion-etched. Unlike the measurements on the degraded and undegraded surfaces where Mg Kα at 1256.7 eV was used for the XPS, Al K_α_ at 1487.6 eV was used instead. This was to enable the Auger transition in the Al KLL at approximately 1395 eV of kinetic energy (KE) to be observed in the XPS spectrum. This corresponds to approximately 92 eV on the *BE* scale. In Figure 4b, the *BE* region between 60 and 140 eV measured at higher resolution is shown. A strong Al 2p plasmon-loss structure is observed [39,40], as expected for a metallic surface. This points to a rather large, unoxidized share of the surface, or rather, to extremely thin Al (of the order of nm) oxide/hydroxide layers in the analysed area, since the intensity of the plasmon-loss structure tends to increase with the metallicity [39,40]. This loss structure overlaps with the Al KLL Auger transition and the Si 2p peak. A differentiated spectrum of the 60–140 eV *BE* region is shown in Figure 4c. Here, the kinetic-energy scale is used. The spectrum was differentiated to better elucidate the Al KLL Auger transition structure and facilitate its comparison with the reference data [38,39,41]. A strong peak at 1395 eV of *KE* is observed. This corresponds to the Al KLL main peak for metallic Al [38,39,41]. There is also a peak of lower intensity at 1389 eV of *KE*, which corresponds to Al in oxide/hydroxide compounds [21,22,37]. The peak at 1380 eV of *KE* corresponds to the second plasmon in the Al 2p loss structure on the *BE* scale (105 eV). The intensity of the peak, however (Figure 4c), is too high for this plasmon only [39,40]. It is due to the overlap of the plasmon with the metallic Al KLL peak, having the second-highest intensity [41]. Thus, an analysis of the Al KLL Auger transition also points to Al oxide–hydroxide formation on the freshly cut surface. In Figure 4d, the narrow-range XPS spectrum for the Al 2p peak is shown fitted with components at 72.0 and 73.9 eV. These correspond to elemental Al and Al oxide/hydroxide [38,39], consistent with the analysis of the Al KLL Auger transition.

#### 3.2.4. Relative Share of Al Oxide versus Al Hydroxide

**Figure 5:**
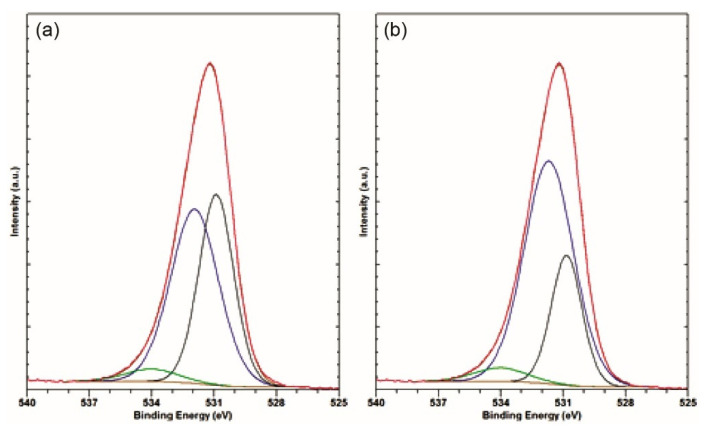
Two different fits of the same O 1s peak using the same components (**a**,**b**).

The oxide layer in the degraded area is very inhomogeneous with some apparently clean spots (Figure 1a), from where most of the Al metal’s signal might derive. While the O 1s peaks can be readily fitted by components corresponding to Al oxide and Al hydroxide with an approximately 1 eV difference, as proposed in [42], two problems arise: several fits of equal quality with different ratios of these components can be obtained, as shown in Figure 5; and, unlike in [42], where Al oxide and hydroxide are supposed to be the only surface compounds, in our sample, there are non-negligible concentrations of other surface compounds, especially silicon oxide and phosphate. The O 1s *BE* values of these compounds overlap with the *BE*s of the Al oxide/hydroxide components.

Therefore, another strategy for estimating the Al oxide vs. Al hydroxide ratio was used. It is an improved and expanded second method from [42], where the ratio of the total Al and O concentrations was used to estimate the oxide/hydroxide ratio. The concentration data from Table 2 were used to estimate the share of the oxygen bound in the phosphates and silicon oxide SiO_x_ with x ≈ 1, which is indicated by Si 2p *BE* ≈ 101 eV. This share was subtracted from the total oxygen concentration to obtain the concentration of Al bound in the oxide and hydroxide, *c*(Al_O,OH_). The ratios *c*(Al_O,OH_)_/_*c*(Al_met_) and *c*(Al_O,OH_)_/_*c*(O_Al_) = α were then calculated from the XPS fitting components’ data in Table 2. The expression *c*(O_Al_) refers to the concentration of oxygen bound in the surface oxide and hydroxide.

The surface oxide/hydroxide composition was supposed to consist of *p*[Al_O,OHO3/2_] and (*1 − p*)[Al_O,OH(OAlH)3_], where *p* and (*1 − p*) are the respective shares of the oxide and hydroxide.

**Figure 6:**
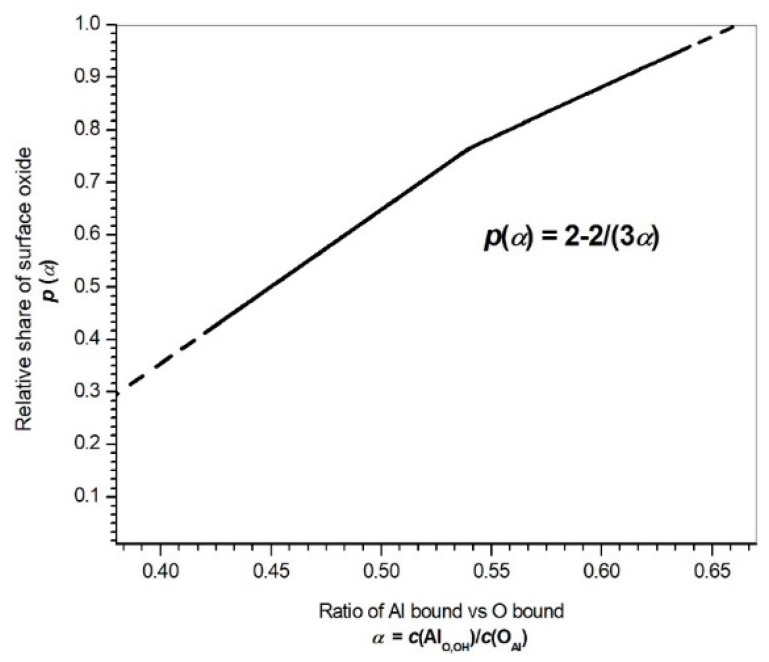
Share of surface oxide *p(α)* vs. ratio of Al and O bound in oxide/hydroxide, *α*.

From this, *c*(Al_O,OH_)/*c*(O_Al_) = *α*(*p*) was calculated, which yielded the oxide share *p*(α) = 2 − 2/(3*α*). This is plotted in Figure 6. It provides a more detailed relation between the stoichiometry and oxide/hydroxide ratio compared to the one in [42]. An overview of the *c*(Al_O,OH_)/*c*(Al_met_), *α* and *p* values obtained is given in Table 3.

The Al hydroxide (*1 − p*) on the freshly cut surface is virtually non-existent (1 − *p* = 0.04), which agrees with previous investigations [43]. It forms, however, a significant part of the oxide/hydroxide layer on the undegraded area and even more significant part of the degraded area (see Table 3).

### 3.3. Surface Roughness

**Figure 7:**
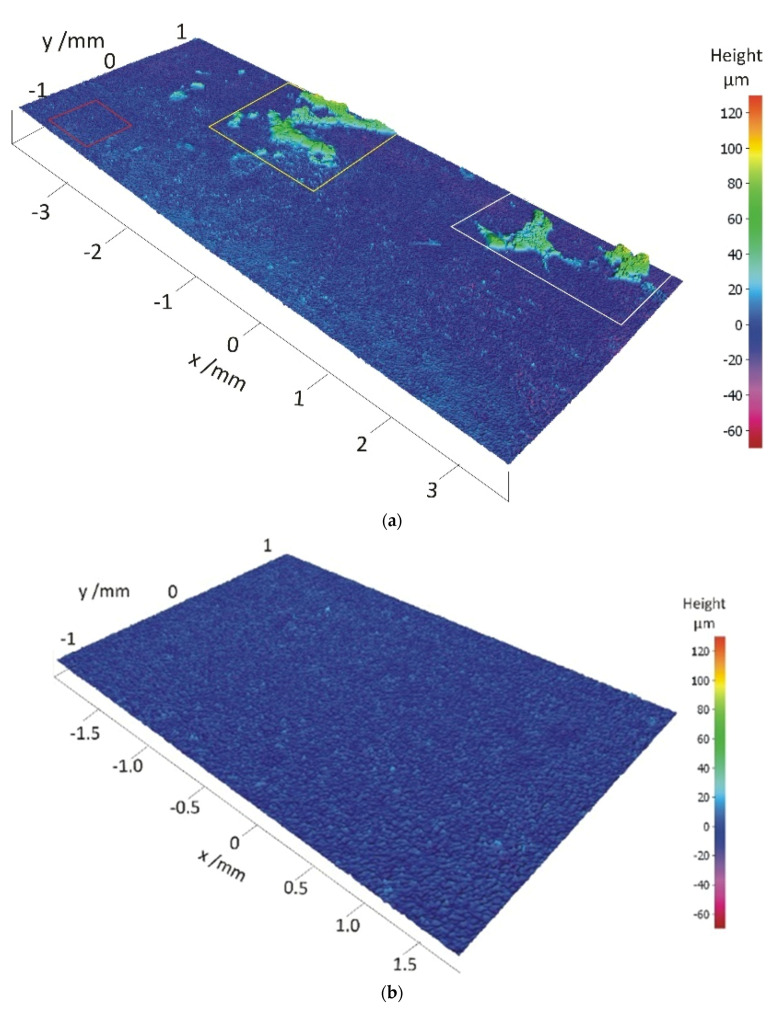

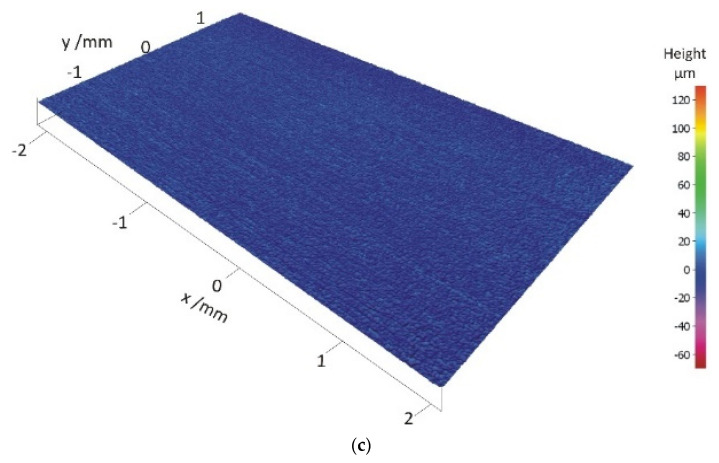
Surface morphology and roughness of the analysed casting’s surfaces: degraded (**a**), undegraded (**b**), and cross-section of the freshly cut casting (**c**).

Surface roughness measurements were performed with a non-contact technique using a 3D optical system to assess the topographical characteristics of the surfaces under investigation. Typical topographies of the degraded and non-degraded samples are shown in Figure 7. It is from these surfaces that the surface parameters were determined. The surface roughness parameters are listed in Table 4.

It can be seen from the topographies and the measured data that the average surface roughness value Sa is the highest in the degraded area (7.09 µm). In contrast to this, the surface roughnesses of the undegraded and the freshly cut samples are approximately three-times lower (2.39 and 2.37 µm, respectively). Accordingly, the Sp and Sv values are much lower here as well (Table 4). A statistical surface roughness parameter skewness (Ssk) describes the distribution of the varying heights. On average, the degraded sample has a positive Ssk value, which is typical of topographies with more (high) peaks than valleys and/or filled valleys. Additionally, selected square areas are highlighted for comparison in Figure 7a (see also Table 4). The selected area at the bottom left in a red square, which shows no signs of degradation, is much more comparable to the undegraded samples shown in Figure 7b,c with respect to the Sa value and a negative Ssk. Negative skewness can be related to deep valleys. However, it should be noted that the values of Sp, Sv, and Sku are much higher than those of the surfaces represented in Figure 7b,c.

## 4. Discussion

The XPS data were used to obtain the surface stoichiometries and information about the compounds present on the degraded and undegraded areas of the Al–Si alloy casting.

Table 2 shows the binding-energy values of the peaks and the atomic concentrations of the elements, derived from the survey and narrow-range spectra. It is interesting to note that a significant concentration of sodium phosphate Na_5_P_3_O_10_ (as estimated from the P concentration) is found in the undegraded, as well as in degraded, area of the sample. This indicates that sodium phosphate itself, from the leftover detergent, does not interact chemically with the surface.

Additionally, the concentration of silicon oxide in the undegraded area is significantly higher than in the degraded area (Table 2). According to the low bulk concentration of Si compared to Al, and Ellingham diagrams of the standard Gibb’s energies of formation for oxides [44], this suggests that silicon oxide is mostly buried under a thicker layer of Al oxide/hydroxide in the degraded area. The Zn 2p peak only appears in the spectra measured on the degraded area, which suggests that Zn precipitation and oxidation on the surface take place only after a sufficiently thick oxide layer is formed.

A calculation using the concentrations of the surface elements and the concentrations of their components in different oxidation states was used to obtain the ratios of the elemental Al vs. Al in the oxide/hydroxide, Al in the oxides/hydroxide vs. O in the oxides/hydroxide, and Al_2_O_3_ vs. Al(OH)_3_. It was found that the share of Al_2_O_3_ was the highest on the freshly cut surface and the lowest on the degraded surface with the thick oxide layer (Table 3).

An initial oxidation of the Al–Si alloys proceeds by an island-by-layer growth mechanism, with an oxide film forming that finally attains a limiting (uniform) thickness [45]. The oxidation is, besides the chemical composition of the alloy, strongly influenced by the temperature and humidity in the surrounding atmosphere [42]. Even ambient-temperature exposures of passivated aluminium to increased relative humidity levels are correlated with unique changes in the passivation layer’s thickness, the thickness uniformity, the crystallinity, and the chemical composition [46]. Similar effects have been reported for the adhesion of unburnt fuel to the piston of a diesel engine made of an Al–Si–Cu alloy [36]. Consequently, the corrosion resistance of the surface of the casting is altered [16,18,19,20,21,36]. However, when the native aluminium oxide containing surface defects that can lead to water adsorption (or absorption) is not sufficient against further corrosion in strong environments, the addition of other passivating agents is recommended [22]. Sol–gel and hydrophobic coatings are also being increasingly used as surface modifications to improve corrosion performance [47,48,49].

The growing trend of using aluminium alloys in the transport industry is driving an increase in the consumption of various aggressive chemicals used in technical cleaning [50]. For example, the prime concern over the use of phosphate detergents is that it increases the phosphorus load on the environment, which, in turn, can lead to problems of eutrophication. Untreated or partially treated effluent will lead to additional phosphorus in surface waters [51].

As an alternative to chemical cleaning procedures, a sustainable cleaning process using dry ice is recommended by Milošev et al. [50]. The dry ice cleaning process is non-invasive to the surface; it requires no use of secondary pollutants (solvents or abrasives), thus ensuring completely dry (waterless) cleaning procedure. It does not leave secondary residues and does not erode or drain the target surface. In contrast, chemical cleaning is more aggressive to the naturally formed protective layer of aluminium and it significantly changes the morphology of the substrate [50].

Based on the results of the present XPS study, we propose that the hygroscopic leftover detergent, in combination with the prolonged time of storage in the sealed plastic envelope, contributes to the onset of the corrosion processes. Storage under condensation causes an aggregation of the water inside the pores of the hydroxide layer [42]. Undoubtedly, the surface condition, i.e., the surface texture of the casting, can play an important role here. The measured values show that the surface roughness value expressed by *Sa* was the highest on the degraded surface (Table 4). Taking into account the negative *Ssk* value, which can mean the presence of deep valleys, e.g., scratches, it can be concluded that the topographic characteristics of this surface enabled better conditions for the cleaning agent’s adsorption. Another consequence of an increased surface roughness is the increase of the active surface area.

The cast aluminium–silicon alloy under investigation is generally used for automobile engine parts, especially cylinder heads [3,4,11,36]. Every loss of structural integrity can lead to serious damage of metallic components [49]. Dimensional tolerance may be lost, and there is a possibility of damage to the material at the contact surface and/or the contamination of the working media [28,52].

In view of the possible roles of both the surface morphology and the humidity, i.e., the water trapped by the hygroscopic leftover detergent as a possible corrosion initiator, it might be advisable to review and reassess the process flow, surface quality, and technical cleaning steps of the castings, mainly with a focus on the better control of the cast topography evolution, cleansing medium, a thorough rinsing process after cleaning, and appropriate packaging conditions.

## 5. Conclusions

An unconventional combination of complementary, surface-sensitive techniques, i.e., XPS and high-resolution 3D optical microscopy, were used as a method for an ex-post material characterization. The degraded and undegraded areas of the Al–Si alloy casting after technical cleaning and packaging were studied. The following conclusions can be drawn:

- The expected Al, Si, and O transitions were observed in the XPS spectra of the degraded surface at binding energies that suggest oxides and hydroxides.
- Hygroscopic sodium phosphate (Na_5_P_3_O_10_) from the leftover cleaning medium was also detected on the degraded and undegraded areas. This phosphate found in the degraded sample could be responsible for the enhanced oxidation by trapping water locally.
- Surface roughness analysis revealed that the topographic characteristics of the degraded surface enabled better conditions for the cleaning agent’s preferential adsorption.
- The Al hydroxide forms a significant part on the degraded area. The share of Al_2_O_3_ is the lowest on the degraded surface with a thick oxide layer and the highest on the freshly cut surface.
- On the freshly cut surface, the presence of Al oxide as well as metallic Al was confirmed. The Al hydroxide is virtually non-existent here.
- The relative shares of oxide/hydroxide on the degraded and undegraded surfaces of the Al–Si casting can be estimated as 3:2 and 3:1, respectively.
- The lower Si-oxide concentration in the degraded area (compared to the undegraded area) suggests that the early-formed Si-oxide layer is buried under a thick Al oxide/hydroxide layer.
- Precipitated Zn from the bulk alloy was only detected on the surface of the degraded area.
- Degradation of the material resulted in a characteristic change of surface dimensions.
- Based on the measurements results, it is recommended to comprehensively re-evaluate the surface quality and the steps of technical cleaning, mainly with a focus on the better control of the cast topography evolution, cleansing medium, a thorough rinsing process after cleaning, and appropriate packaging conditions.
- An alternative to chemical cleaning procedures, a sustainable cleaning process using dry ice can be advised [50].
- Surface-sensitive techniques such as XPS and an optical 3D surface measurement system act as suitable analytical tools to identify the source of the defects on the cast components. They can be effectively used in the design of foundry processes.

## Figures and Tables

**Table 1:** Chemical composition of the A380 bulk alloy in wt%.

Content	Si	Cu	Zn	Mn	Fe	Mg	Sn	Ni	Al
*c*/wt%	9.4	3.80	3.0	0.10	1.25	0.1	0.30	0.4	Bal.

**Table 2:** XPS binding-energy values and the surface chemical composition according to the survey and high-resolution XPS spectra of the degraded area, the undegraded area, and the area on the freshly cut surface.

Sample	Degraded		Undegraded		Freshly Cut Alloy	
Component	BE/eV	c/at%	BE/eV	c/at%	BE/eV	c/at%
O 1s	531.5	47.41	531.5	26.07	531.5	38.50
N 1s	399.74	1.41	399.89	2.38	-	-
Na 1s	1071.86	0.53	1072.0	3.34	-	-
C 1s	284.73	26.15	284.9	50.44	284.8	14.88
Al 2p 1	74.20	15.04	73.9	6.62	73.90	24.67
Al 2p 2	72.67	3.49	72.3	2.44	71.9	20.28
P 2p 1	130.50	0.46	130.39	0.29	-	-
P 2p 2	132.69	3.39	133.04	3.33	-	-
Zn 2p_3/2_	1021.77	0.31	-	-	-	-
Si 2p 1	101.89	0.81	101.90	3.85	-	-
Si 2p 2	100.52	0.88	100.39	0.93	-	-
Ca 2p	346.89	0.11	347.17	0.33	-	-

**Table 3:** Calculated surface composition regarding shares of Al bound in the oxide and hydroxide vs. Al_met_, ratio of Al_bound_ in the oxide and hydroxide vs. oxygen bound in those compounds and the Al_2_O_3_ in Al oxides and hydroxides (*p*).

Relative Share	Al_bound_/Al_met_	(Al/O)_bound_	Oxide	Hydroxide
Surface	c(Al_O,OH_)/c(Al_met_)	*α*	*p*	*1 − p*
Degraded area Degraded	4.31	0.42	0.58	0.42
Undegraded area	2.71	0.54	0.77	0.23
Freshly cut area	1.22	0.64	0.96	0.04

**Table 4:** Surface roughness measurements.

	Sample					
	Degraded				Undegraded	Fresh-Cut
Area	7.7 × 3.1 mm^2^	Red Square	Yellow Square	White Square	3.1 × 2.0 mm^2^	4.8 × 2.7 mm^2^
Parameter		Bottom Left	Upper Left	Upper Right		
Sa/µm	7.09	3.17	17.21	16.92	2.39	2.37
Sq/µm	11.51	4.35	21.71	22.18	3.12	3.04
Sp/µm	110.07	42.38	102.17	115.38	28.40	15.80
Sv/µm	63.87	42.73	60.32	55.39	23.05	22.67
Sz/µm	173.94	85.12	162.49	170.76	51.45	38.46
S10z/µm	160.46	72.74	153.91	161.32	43.64	35.75
Ssk	2.24	−0.51	1.28	1.65	−0.14	−0.08
Sku	12.58	8.1813	4.01	5.03	4.53	3.58

## Data Availability

The data used to support the findings of this study are available from the corresponding author upon request.

## Abbreviations

The following abbreviations are used in the manuscript:

Ar^+^	Argon ion
Al_bound_	Al bound
Al_met_	Metallic Al
Al_O,OH_	Al bound in in the oxide and hydroxide
Al_2_O_3_	Aluminium (III) oxide
Al(OH)_3_	Aluminium hydroxide
*α* = *c*(Al_O,OH_)/*c*(O_Al_)	Ratio of (Al/O) bound in the surface oxide and hydroxide
BE	Binding energy
*c*(Al_O,OH_)	Concentration of Al bound in the oxide and hydroxide
*c*(O_Al_)	Concentration of oxygen bound in the surface oxide and hydroxide
ICP-OES	Inductively coupled plasma optical emission spectroscopy
KE	Kinetic energy
Na_5_P_3_O_10_	Sodium phosphate
O_Al_	Oxygen bound in the surface oxide and hydroxide
*p*	Surface share of oxide
*p*[Al_O,OHO3/2_]	Assumed composition of surface oxide
(*1 − p*)	Surface share of hydroxide
(*1 − p*)[Al_O,OH(OAlH)3_]	Assumed composition of surface hydroxide
Sa	Average height of selected area
Sq	Root–Mean–Square height of selected area
Sp	Maximum peak height of selected area
Sv	Maximum valley depth of selected area
Sz	Maximum height of selected area
S10z	Ten-point height of selected area
Ssk	Skewness of selected area
Sku	Kurtosis of selected area
XPS	X-ray photoelectron spectroscopy
